# Maternal and Neonatal Vitamin D Status at Preterm Delivery—A Cross-Sectional Study

**DOI:** 10.3390/nu18172908

**Published:** 2026-09-04

**Authors:** Magdalena Zarlenga, Ewa Głuszczak-Idziakowska, Justyna Czech-Kowalska, Michał Skrzypek, Maria Wilińska

**Affiliations:** 1 Clinical Department of Rare Diseases, Neonatal Intensive Care and Therapy, University Teaching Center, Medical University of Warsaw, 02-091 Warsaw, Poland; 2 Department of Neonatology, The Medical Centre of Postgraduate Education, 01-813 Warsaw, Poland; 3 Department of Neonatology and Neonatal Intensive Care, The Children’s Memorial Health Institute, 04-730 Warsaw, Poland; j.kowalska@ipczd.pl; 4 Department of Biostatistics, Public Health Department in Bytom, Medical University of Silesia, 41-902 Katowice, Poland; mskrzypek@sum.edu.pl; 5 Department of Neonatal Physiology, Pathology and Intensive Care, National Medical Institute, The Ministry of the Interior and Administration, 02-507 Warsaw, Poland

**Keywords:** vitamin D, 25OHD, prematurity, very low birth weight (VLBW), preterm labor, pregnancy

## Abstract

Background: An optimal 25OHD level can prevent osteopenia of prematurity (bone health) and premature delivery (non-skeletal activity). Although maternal and fetal vitamin D (25OHD) levels correlate, data on preterm infants’ 25OHD status at birth are limited, with a well-recognized deficiency among term infants. Objectives: To evaluate maternal and neonatal 25OHD status at preterm birth and its determinants in a northern-latitude country, Poland. Design/Methods: Cross-sectional study between August 2015 and September 2016 investigating cord and maternal blood 25OHD levels at delivery in 69 pairs of mothers and newborns ≤ 32 6/7 weeks of gestation, and analyzing correlations between maternal and neonatal 25OHD levels and their influencing factors, with univariable and multivariable linear regression. Results: Median (IQR) 25OHD levels were 25 (16.6–34 ng/mL) in cord blood and 21.2 (15–29 ng/mL) in mothers and correlated positively (r = 0.69, *p* < 0.0001). 25OHD insufficiency was detected in 84.06% of mothers and 62.32% of newborns. Maternal 25OHD levels were higher in summertime delivery (*p* = 0.0008). The rate of vitamin D supplementation during pregnancy was 54.4%, increasing maternal (27 ng/mL vs. 16.7 ng/mL, *p* = 0.0006), cord blood (32.4 ng/mL vs. 17.4 ng/mL, *p* = 0.0005), and summer seasonal cord blood 25OHD levels (38.89 ng/mL vs. 25.5 ng/mL, *p* = 0.023), and the percentage of maternal (72.7% vs. 27.3%, *p* = 0.005) and neonatal 25OHD sufficiency (80% vs. 20%, *p* = 0.0021). Conclusions: Vitamin D insufficiency is common in mothers and their prematurely born infants at birth; vitamin D supplementation reverses the trend. To avoid inadequate supplementation in preterm newborns and in mothers at risk of preterm delivery, regular assessment of 25OHD levels should be considered.

## 1. Introduction

Vitamin D status influences general health. The function and regulation of several tissues and organ systems in the human body depend on this status [1].

Low 25-hydroxyvitamin D (25OHD) level in pregnancy increases the risk of pre-eclampsia, gestational diabetes mellitus, and preterm labor [2,3,4,5,6]. Gestational 25OHD > 40 ng/mL is associated with a 60% lower risk of premature birth [7]. Insufficient maternal vitamin D [8] and preterm interruption of transplacental transport of vitamin D, calcium, and phosphorus increase the risk of osteopenia of prematurity and vitamin D deficiency in infants, especially those born prematurely [9]. Vitamin D status in pregnancy affects the development of fetal lungs and brain, bone health, immune response, and the prevalence of respiratory tract diseases in offspring later in life [10,11,12]. Adequate vitamin D stores have a confirmed impact on antibacterial defense [13], pulmonary and cardiovascular system function [14,15], and iron turnover [16]. It makes investigating the scale and causes of vitamin D deficiency particularly important in preterm infants <32 6/7 weeks of gestational age.

Serum 25OHD level is used as an indicator of vitamin D status [17,18]. Vitamin D status is defined as deficiency for levels <20 ng/mL, insufficiency (suboptimal level) for levels 20–30 ng/mL, and sufficiency for levels >30 ng/mL [19].

A level > 20 ng/mL, according to the latest recommendations for preterm and very-low-birth-weight (VLBW) infants, determines the optimal level for proper bone mineralization [20,21]. However, a level ≥ 30 ng/mL ensures nonskeletal benefits for stable, growing preterm infants [18,22]. A higher risk of hypercalcemia exists in preterm infants with a 25OHD level above 80 ng/mL [23]. The latest Polish recommendations determine the optimal 25OHD level as 30–50 ng/mL for the whole population [24].

Vitamin D insufficiency among healthy pregnant women can reach up to 95% [25,26]. Gestational vitamin D status depends on vitamin D supplementation during pregnancy and skin synthesis [27,28,29]. Fetal and neonatal vitamin D status at birth strongly depends on maternal vitamin D stores during pregnancy [9,17,30,31]. 25OHD maternal and umbilical cord blood levels correlate positively [26,27,29,31,32,33]. Higher vitamin D intake may be necessary for preterm infants after birth to normalize decreased 25OHD levels caused by prenatal vitamin D deficiency [6,9,22,24].

About 50% of full-term newborns have vitamin D insufficiency at birth [17,34,35], but there is a shortage of studies in preterm infants [36]. Vitamin D insufficiency is likely to be more frequent among premature infants [29,32,33,34,37]. The documented prevalence of 25OHD insufficiency/deficiency at preterm birth was about 40%, and the majority of neonates have sufficient vitamin D levels at six to eight weeks of life at the time of discharge [9]. Given the significant proportion of preterm neonates who are deficient/insufficient at birth, with a significant proportion of neonates still being deficient/insufficient at 3 to 4 weeks of life, there is a potential role for 25OHD screening at birth to ensure adequate vitamin D supplementation and decrease serious consequences later in life [9].

The study aimed to evaluate 25OHD concentrations in premature infants and their mothers to determine overall vitamin D status and its clinical predictors.

## 2. Materials and Methods

Institutional Review Board permission was obtained (25 February 2015) to enroll pairs of Caucasian mothers and infants born at <32 6/7 weeks gestation (WG) in the research project entitled “Factors affecting calcium and phosphate homeostasis and vitamin D level in newborns born < 32 weeks gestation and their mothers”, registered at ClinicalTrials.gov (NCT03691895). Parental consent was obtained in each case. The first part of the project was a cross-sectional study performed between 31 August 2015 and 21 September 2016 in the neonatal intensive care unit of the Centre of Medical Postgraduate Education in Poland. The inclusion criterion was labor at <32 6/7 WG, and the exclusion criteria were labor at ≥33 0/7 WG or the presence of any congenital defects.

Data were collected from interviews with mothers and maternity records and recorded via an electronic questionnaire. Maternal and neonatal demographic and clinical data included age, weeks gestation, birth weight (BW) (g), BW percentile values [38], maternal BMI (body mass index, kg/m^2^) before pregnancy and on the day of labor, weight gain during pregnancy, Apgar score at the 5th minute of life, sex, season of labor, maternal illnesses (thyroid diseases, viral or parasitic infections, smoking, alcohol use, diabetes, epilepsy, asthma, cardiovascular problems), medications, prenatal steroid therapy for lung maturation, prenatal supplementation with calcium, docosahexaenoic acid (DHA), and vitamin D intake. For each participant mother, data regarding supplement duration, dosage, and formulation type—categorized as vitamin D alone, vitamin D with DHA, or multivitamin preparations—were recorded.

The WG was determined by an ultrasound performed between the 10th and 12th weeks of pregnancy. Birth weight < 10th percentile defined intrauterine growth restriction (IUGR) [38].

Pregnant women who reported taking vitamin D supplements during pregnancy were assigned to the “supplementing mothers” study group, irrespective of supplement duration or formulation. To evaluate the impact of supplementation, maternal and neonatal 25OHD concentrations on the day of birth were analyzed and compared between mothers who did and did not take vitamin D supplements. The subgroups of neonates and mothers within the supplemented cohort were categorized based on the duration of supplementation and three distinct maternal dosage metrics: total cumulative dose, mean dose, and mean daily dose.

To investigate the impact of insolation on vitamin D synthesis in Poland, summertime deliveries were defined as those occurring between 1 June and 30 September, and wintertime deliveries between 1 October and 31 May [39].

Total 25OHD levels were measured in maternal and umbilical blood samples collected on the day of labor. The electrochemiluminescence immunoassay (ECLIA, Roche, Switzerland, Cobas e411), consistent with the ID-LC-MS/MS reference method for 25-hydroxyvitamin D [17], was performed within 24 h after delivery. The assay method on the ELECSYS system utilizes ruthenium-labeled vitamin D-binding protein (DBP). According to the manufacturer’s data, its cross-reactivity with 25OHD3, 25OHD2, 24,25(OH)_2_D3, 1,25(OH)_2_D3, 1,25(OH)_2_D2, vitamin D3, vitamin D2, and the 25OHD3 C3-epimer is 98%, 81%, 121%, 5%, 6%, 5%, 6%, and 93%, respectively. The limit of detection is 3 ng/mL (7.5 nmol/L). The limits of quantitation represent the lowest concentration of the analyte that can be determined with a precision (CV) of <20%.

Definition of 25OHD status:

The definition was based on clinical international and national practice guidelines (Endocrine Society, ESPGHAN and others [19,40,41]).

Levels > 10–20 ng/mL determined deficiency (with ≤10 ng/mL classified as severe deficiency), levels > 20–30 ng/mL determined insufficiency (suboptimal level), and levels > 30 ng/mL determined sufficiency.

Accordingly, based on vitamin D status, three study groups were established for both, mothers and for newborns.

Compatibility between the distribution of quantitative variables and a normal distribution was verified using the Shapiro–Wilk test. For comparisons between two or more groups, the Mann–Whitney test and Kruskal–Wallis test were used. Dependence between two quantitative variables was assessed with a univariate linear regression model, and the Spearman’s rank correlation coefficient was also calculated. Multivariable linear regression was used to estimate the coefficient β with 95% confidence intervals and *p*-values. The univariable model was fitted first, then variables with *p* ≤ 0.3 were imputed into the multivariable model. The stepwise backward elimination method with the Akaike criterion was used to derive the final model. Multicollinearity was checked by means of the VIF (Variance Inflation Factor) and correlation analysis between explanatory variables. Demographic and clinical data are presented as mean, standard deviation (SD), and standard error, or median and Interquartile Range (IQR), as appropriate. Categorical variables are presented in absolute terms and as percentages. We assumed that data from 69 pairs (mother–child) would provide sufficient statistical power to detect meaningful differences.

All statistical analyses were performed utilizing SAS version 9.4 (SAS Institute Inc., Cary, NC, USA). A *p*-value of <0.05 was considered statistically significant. The sample size estimation was performed using the pwr.f2.test function from the pwr package in R software version 4.6.1 (R Foundation for Statistical Computing, Vienna, Austria).

## 3. Results

During the study period, 73 mother–infant pairs met the inclusion criteria, and final statistical analysis covered 69 of them. Two pairs were not included due to lack of maternal consent, and two were excluded due to unavailable blood samples.

The required sample size was calculated for the multivariable linear regression model. We assumed a statistical significance level (α) of 0.05, a power of 0.80, and an expected proportion of variance explained by the model (R2) of 0.20, which corresponds to a Cohen’s effect size (f2 = 0.25). The minimum required sample size was determined to be 65 observations, assuming 7 explanatory variables.

### 3.1. Clinical Characteristics

The mean gestational age of 69 preterm infants was 28.8 ± 2.6 weeks, mean birth weight was 1221 g ± 416, and mean body weight percentile was 51.8 ± 26.8. More infants, 44 (63.8%), were born in the winter season. The detailed clinical neonatal and maternal characteristics are presented in Table 1.

Among the mothers participating in the study, diabetes was diagnosed in only two and asthma in one; none of the subjects were found to have other health issues mentioned in the questionnaire or coming from official medical records.

### 3.2. Maternal and Neonatal Vitamin D Status

**Figure 1:**
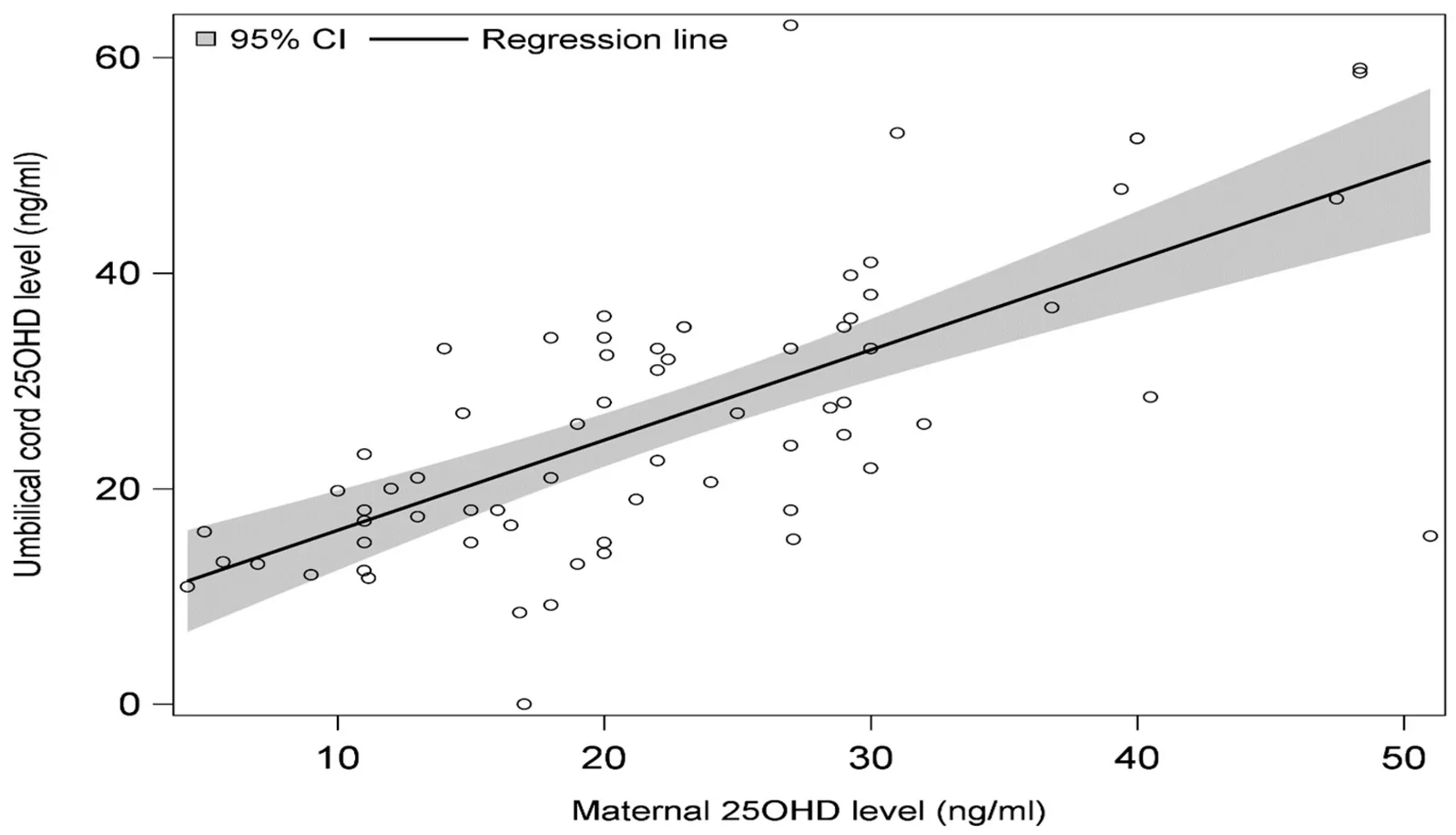
Scatter plot showing the correlation between maternal 25OHD levels and umbilical cord 25OHD levels (r = 0.69; *p* < 0.0001. Each point represents an individual maternal-fetal pair. The solid line indicates the linear regression line, and the shaded area represents the 95% confidence interval (CI).

Maternal and neonatal cord blood 25OHD levels demonstrated a strong positive correlation (*r* = 0.69; *p* < 0.0001; Figure 1). The median cord blood 25OHD level was significantly higher than the maternal concentration [21.2 (IQR: 4.36–51.00) vs. 25.0 (IQR:0.00–63.00) ng/mL, respectively; *p* < 0.0001].

Optimal vitamin D status (25OHD: 30–50 ng/mL) was observed in 37.68% of newborns and 15.94% of mothers, while vitamin D deficiency was diagnosed in 40.6% of mothers and 69.7% of newborns (Table 2). The rate of severe vitamin D deficiency (25OHD < 10 ng/mL) was relatively low in both groups, affecting 7.25% of mothers and 4.4% of newborns.

### 3.3. Vitamin D Status and Season

Maternal 25OHD levels differed significantly across seasons, with markedly higher values observed in summer than in winter (Table 1). The prevalence of vitamin D sufficiency among mothers who delivered during the summer was 72.7%, compared to 27.3% for those with winter deliveries. Indeed, the lowest percentage of maternal vitamin D deficiency (17.9% vs. 82.1%) was noted in the summer season (Table 2).

**Figure 2:**
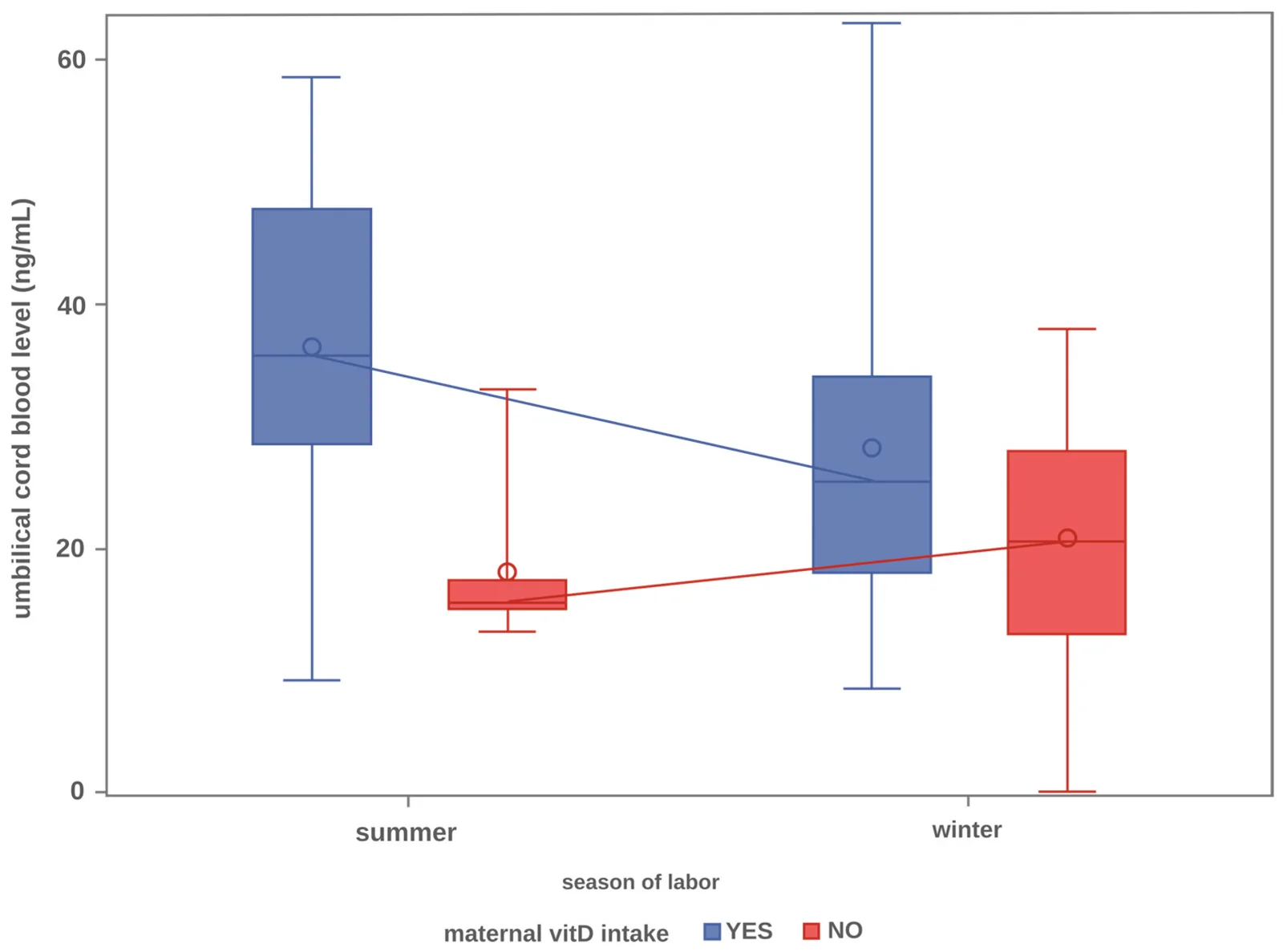
Comparison of the effect of birth season on umbilical cord 25OHD levels between subgroups of newborns whose mothers were either supplemented or not supplemented with vitamin D. ° Arithmetic mean.

A significantly higher cord blood 25OHD level was recorded in the subgroup of summer births compared to winter births (median [IQR]: 38.89 [19.3] ng/mL vs. 25.5 [16.0] ng/mL; *p* = 0.023). However, these elevated cord blood 25OHD levels were associated with both summer delivery and maternal vitamin D supplementation during pregnancy (Figure 2).

### 3.4. Vitamin D Status and Maternal Vitamin D Supplementation During Pregnancy

Higher neonatal and maternal 25OHD levels in cases where mothers reported vitamin D supplementation during pregnancy (Table 1). The prevalence of vitamin D sufficiency was higher among supplemented mothers (72.7%) and their preterm offspring (80%) compared with non-supplemented mothers (27.3%) and their offspring (20%) (*p* = 0.0021).

The highest daily vitamin D intakes during pregnancy (1180IU; IQR: 655.24–1400) were reported among mothers with serum 25OHD level > 30 ng/mL.

The minimum duration of vitamin D supplementation was 2 weeks and maximum 20 weeks.

The mean total amount of vitamin D intake throughout pregnancy was (156,578 ± 110,275 IU) and correlated positively with the umbilical 25OHD level (R = 0.31; *p* = 0.06).

The median daily vitamin D intake was 1003.13 IU (IQR: 795.54 IU). It correlated significantly with maternal (r = 0.5; *p* = 0.002) but not with cord 25OHD levels (r = 0.27; *p* = 0.11).

A vitamin D intake per day per mother of 769.15 IU [87.56–2000] correlated with maternal 25OHD level (r = 0.26; *p* = 0.0013) but not with cord blood 25OHD level (r = 0.05; *p* = 0.1). Conversely, the duration of supplementation showed no significant association with the vitamin D status of either the mother (*p* = 0.11) or child (*p* = 0.93).

Vitamin D intake was based on widely available dietary supplements (single or multi-component supplements allowed for pregnant women). DHA supplementation during pregnancy was associated with higher maternal and cord blood 25OHD levels (Table 1). Most mothers (52%) used DHA, and usually with vitamin D. We noted only one mother who supplemented vitamin D without DHA.

Among the 57 mothers in the overall cohort, those who received antenatal corticosteroid therapy for fetal lung maturation and were in the “supplementing group” delivered infants with neonatal vitamin D sufficiency significantly more frequently than non-supplementing mothers [18 (82%) vs. 4 (18%), *p* = 0.0014]. However, no significant association was observed between the administration of prenatal steroid therapy and cord blood 25OHD concentrations (no: 12 (17.4%) vs. yes: 57 (82.6%), *p* = 0.37).

### 3.5. Independent Predictors of Neonatal and Maternal Vitamin D Status at Preterm Delivery

Univariate linear regression analysis was conducted to identify factors influencing cord blood and maternal 25OHD levels. In the analysis targeting cord blood 25OHD as the outcome variable (Table 3), maternal 25OHD concentration emerged as a highly significant predictor (β = 0.837, 95% CI: 0.616 to 1.058, *p* < 0.0001). Similarly, maternal vitamin D supplementation status (maternal supplementation Vitamin D_[yes/no]) showed a strong positive association (β = 11.536, 95% CI: 5.879 to 17.193, *p* = 0.0001). Seasonal factors (*p* = 0.0635) and maternal DHA levels (*p* = 0.0612) exhibited borderline significance. Other examined maternal and neonatal baseline characteristics, including birth weight, Apgar score, and gestational age, did not demonstrate statistically significant associations in the univariate models (all *p* > 0.05).

No statistically significant association was found between cord blood 25OHD concentration and the other parameters we examined: maternal pre-pregnancy and delivery-day BMI, change in maternal BMI up to the time of delivery, pregnancy order, maternal age, hypothyroidism, and hypertension (Table 3). It was not possible to assess the impact of diabetes, asthma, or hyperthyroidism on cord blood 25OHD concentration in this study due to the insufficient number of affected mothers.

Multiple linear regression analysis (Table 4) revealed that neonatal 25OHD levels were independently predicted by maternal serum 25OHD levels, maternal vitamin D intake, and male infant sex. The model was statistically significant, F(3.64) = 22.96, *p* < 0.001 and adjusted R^2^ = 0.50.

## 4. Discussion

A high prevalence of vitamin D deficiency in both mothers and their neonates was found along with a strong positive correlation between 25OHD serum levels in mothers and in umbilical cord blood, similar to previous reports [26,27,28,42]. However, low maternal vitamin D status strongly predicted low vitamin D levels in their newborns, as confirmed by other researchers [26,32,42,43]. Our findings align with previous research [42] demonstrating that maternal 25OHD status is the primary determinant of neonatal vitamin D levels, outweighing fetal genetic and biological factors.

Consequently, over 60% of the newborns had vitamin D insufficiency in our cohort, which equals a real risk for stable growth and bone health of VLBW neonates [22,44]. Insufficiency was defined as levels below the norm (25OHD level < 30 ng/mL) but above the critical threshold (>20 ng/mL). Indeed, a 25OHD level > 20 ng/mL represents a threshold that persistently elevates the risk of future skeletal mineralization deficits [45]. A potential limitation of our study is that the assay method used for vitamin D determination [41,46] may have overestimated the proportion of neonates with at least sufficient 25-OHD levels. In our study, in cases of maternal vitamin D deficiency, only every third infant managed to reach vitamin D insufficiency. Crucially, our findings pertain specifically to the cohort of preterm infants. Nevertheless, no neonatal sufficiency occurred among the 10% of mothers who delivered prematurely and exhibited 25OHD levels < 10 ng/dL. These findings parallel studies from the USA, Australia, Korea [5,9,25,26], and Poland [6]. The Polish study demonstrated 25OHD levels < 10 ng/mL as representing an increased risk of preterm labor [6]. Other studies point to adequate maternal vitamin D levels during pregnancy as critical for general health in the future [2,20,21].

In contrast to observations by Czech-Kowalska [27] of term mother–infant pairs in the same Polish region, and to European VLBW cohorts [47], our study observed higher maternal and neonatal vitamin D levels and a lower prevalence of deficiency. Unfortunately, the results are always unsatisfactory and present a higher risk for ex-preterm children in the future [44,45].

In the USA, vitamin D deficiency concerns 25–64% of preterm newborns born < 32nd WG [43,48], similarly to the 40% in the present study. Burris et al. [48] showed insufficient vitamin D status in 40% of term and preterm newborns. Similarly to this study, they found no clear association between gestational age and umbilical blood 25OHD level. However, more immature preterm newborns (<32 GA) had a 2.5 times higher risk of vitamin D deficiency. Monangii et al. [43] observed a higher degree of vitamin D deficiency in children born < 28th WG (almost 70%) than between 28th–32nd WG (50%). The risk was twice as high if children were born < 28 WG than between 28th and 32nd WG.

In our study, only 16% of non-supplemented mothers and 35% of supplemented mothers had sufficient vitamin D status. The maternal supplementation increased the newborn’s likelihood of achieving a 25OHD level > 30 ng/mL at birth—a state representing sufficient vitamin D stores—to over 80%. This study found that higher maternal vitamin D intake improved both maternal and neonatal vitamin D levels at birth. A daily intake of ~1000 IU, as noted, significantly correlated with sufficient maternal levels (*p* = 0.002). The lack of a direct significant correlation with cord blood (*p* = 0.11) at this specific dosage may be elucidated by reviewing alternative literature in this field. Vitamin D intake during pregnancy improves 25OHD maternal–fetal transfer via the placenta [4,12,27,49]. However, the fetus relies entirely on the mother’s total circulating vitamin D reservoir, which is heavily influenced by her baseline levels, genetics, BMI, and sun exposure—not just her daily pill intake [50].

The difference in maternal–fetal vitamin D status found in this study might be explained by the higher recommended doses implemented in Poland since 2013, which consist of a daily intake of 1500–2000 IU throughout pregnancy [24,44]. Nevertheless, only half of the surveyed pregnant women confirmed consistent vitamin D intake.

A cohort study performed in Norway showed that daily vitamin D supplementation at a dose of 600–800 IU reduced the risk of preeclampsia by 31%. Simultaneously, the researchers demonstrated that diet did not supply an adequate amount of vitamin D [4,51]. We could not precisely evaluate the dietary vitamin D intake during pregnancy. However, none of the participating mothers reported following a vegetarian or vegan diet, and dietary products were not limited. Additionally, regular consumption of sea fish is generally low in the general Polish population [51].

In this study, a group of mothers utilizing concurrent DHA and vitamin D supplementation exhibited significantly elevated 25OHD concentrations. Achieving similar outcomes, Norwegian and other groups proposed that a mutual positive interaction between DHA and 25OHD may enhance maternal vitamin D bioavailability [4,52]. Given that dietary DHA intake is characteristically high within the Norwegian population, a 2009 study involving a cohort of nearly 25,000 pregnant women demonstrated that 25OHD concentrations remained significantly lower among women who did not use vitamin D supplements [4,44].

Finding no association of umbilical 25OHD level with the duration of supplementation (*p* = 0.11) is also biologically plausible. A notable finding in our study is that total cumulative vitamin D intake throughout pregnancy demonstrated a stronger trend toward neonatal cord blood sufficiency (*r* = 0.31; *p* = 0.06) than either daily dosage or supplementation duration analyzed independently. Vitamin D levels often plateau after a few weeks of consistent dosing, meaning taking it for 20 weeks might not result in a higher level than taking it for 12 weeks [53]. Understanding the regulatory mechanisms and rate-limiting steps in the relationship between vitamin D and the placenta is still debated [54]. From a pharmacokinetic perspective, this suggests that fetal vitamin D accumulation is determined by the total maternal reservoir built up over the course of gestation rather than isolated daily peaks [41,55].

The mothers who underwent antenatal two-dose corticosteroid therapy and supplemented with vitamin D delivered vitamin D-sufficient infants significantly more often compared to non-supplementing mothers. We suppose first that the simple positive effect is due to prenatal vitamin supply. Obviously, as corticosteroids upregulate the expression of the vitamin D receptor (VDR) and modulate key cytochrome P450 enzymes within the placental trophoblast, it could optimize the local conversion and placental transfer of 25OHD to the fetus [54,56].

In the studied group, the umbilical cord blood 25OHD level was significantly higher than the maternal level, as also reported in the German population [29]. However, not all infants exhibited higher umbilical cord blood 25OHD levels compared to their mothers. However, a false-positive effect on neonatal vitamin D status of the laboratory method is highly likely due to overestimation of the result caused by counting the C3-epimer of vitamin D [17,24,41,46].

Interestingly, we observed variations in neonatal concentrations among individual infants despite relatively similar maternal levels. This discrepancy may be influenced by the placental transfer mechanism of vitamin D [54,55,56]. Crucially, the complex metabolism and distinct physiological roles of individual vitamin D metabolites during pregnancy are still being examined. Placental transport primarily involves 25OHD. The human endometrium produces 1,25(OH)2D and 24,25(OH)2D, whereas the placenta actively synthesizes 24,25(OH)2D [57].

Elevated results of 25OHD in newborns could be attributed to the co-measurement of 3-epi-25 hydroxyvitamin D (3-epi-25OHD). Higher levels of 3-epi-25OHD in umbilical cord blood (ranging from 4% to 22%) than in adults might be the reason. 3-Epi-25OHD mineral activity remains weaker, and its biological role is still not well known [17,28,46].

Consistent with previous findings [27,47], we observed a significant association between higher maternal vitamin D status and summertime labor. As argued by Krzyścin [39], this highlights the important role of endogenous skin synthesis, particularly in sunny regions or during the summer in northern latitudes. Conversely, no direct association was found for infants, aligning with the results of Lawlor et al. [58]. However, a significant association emerged for infants when combining summertime birth with active vitamin D intake. Additionally, this relationship highlights that without maternal supplementation, even high seasonal sun exposure cannot guarantee adequate vitamin supply for the newborn [8]. Moreover, endogenous skin synthesis of 25OHD is reduced by 90–95%, with dermatological recommendations to use sunscreens during insolated months [24]. Consequently, vitamin supply is necessary year-round [24,44]. This leads us to hypothesize that standard winter vitamin D supplementation cannot fully compensate for the lack of natural insolation [41].

Low vitamin D status in our study, observed in more than half of the preterm infants born ≤ 32 6/7 WG, needs special attention. Severe vitamin D deficiency at birth requires high vitamin D intake to improve vitamin D status [22]. Munshi et al. [59] reported low vitamin D status in 80% of VLBW infants at 4 weeks of age, although they received recommended vitamin D supply or higher [9,20,36]. Following oral vitamin D supplementation, 25OHD levels increased significantly in VLBW infants by weeks 8 and 12 of age. In contrast, extremely-low-birth-weight (ELBW) infants lagged at week 8, demonstrating a significant increase only by 12 weeks of age [59]. Establishing baseline 25OHD levels at birth could allow for optimized, individual dosing from the onset of supplementation, thereby avoiding hypovitaminosis or inadvertent overdose [60]. This approach strongly supports the trend toward personalized medicine in neonatology. Nevertheless, despite the high vulnerability of very preterm infants (<32 6/7 weeks of gestation), routine monitoring at birth is absent from current guidelines, with the first screening generally delayed until after four weeks of supplementation [24,44,61].

At present, there are various recommendations for vitamin D supplementation in preterm newborns, and recommended daily doses range from 400 IU to 1000 IU [20,22,61,62,63]. Assessment of maternal and cord 25OHD levels may optimize vitamin D administration during pregnancy and in the first days of life in preterm infants, and avoid negative outcomes in late infancy and early-school age [23,45].

The present study addresses a clinically relevant research question by focusing on a highly vulnerable preterm population, an area where data regarding micronutrient optimization remain limited. Using umbilical cord blood for research reduced potential iatrogenic blood loss and enabled the measurement of neonatal vitamin D levels on the day of birth. A major strength of our protocol is the simultaneous assessment of maternal and cord blood vitamin D status to investigate the maternal–fetal transfer dynamics in premature births.

This study has several limitations that warrant consideration. Firstly, this was a small, single-center observational cohort with limited generalizability. Therefore, we could not hypothesize that the vitamin D profile of the pregnant women participating in this study reflects the broader Polish population. However, other recent multicenter studies from Poland have documented highly insufficient vitamin D status among pregnant women [24,44]. Moreover, the evaluation of prenatal vitamin D intake or supplementation and sunlight exposure relied on a retrospective analysis. To mitigate recall bias, maximum care was taken during data collection by cross-referencing patient interviews with official maternity records to confirm exact dosages and supplementation durations, and compliance with supplementation. We were aware that the participating mothers utilized a wide variety of prenatal vitamin D formulations with highly heterogeneous cholecalciferol contents, which were rarely recognized as actual medicinal products, thereby complicating standardized quantification. Furthermore, dietary vitamin D intake was not strictly assessed; nonetheless, nutritional intake of vitamin D was documented to be consistently low in both Poland and other European countries [8,51]. Finally, other maternal confounding variables potentially influencing newborn birth weight—such as overall dietary quality and total caloric intake, maternal socioeconomic status, and physical activity—were not accounted for in the current study design.

Another methodological limitation is the use of an automated immunoassay (ECLIA) for routine 25OHD testing instead of the chromatographic “gold standard” (LC-MS/MS) [SRM 972; Vitamin D in Human Serum. National Institute of Standards and Technology (NIST): Gaithersburg, Maryland, USA, 2009]. Both during the study period and a decade later, during the drafting of this manuscript, LC-MS/MS remained a method available only in selected specialized centers. Although international guidelines favor assays measuring both 25OHD2 and 25OHD3 [17,41], advanced methods ideally exclude the 3-epi-25OHD3 metabolite. Because automated platforms are inherently susceptible to cross-reactivity with this C3-epimer, a potential overestimation of total 25OHD levels in our newborn cohort cannot be entirely ruled out. Nevertheless, ECLIA remains a highly reliable, widely accepted tool in routine clinical practice, with an intra-assay coefficient of variation well within acceptable diagnostic thresholds.

Despite these limitations, our findings demonstrate that vitamin D deficiency remains a prevalent and critical health concern among mothers and their preterm offspring. Crucially, this high prevalence persists even though clear national guidelines regarding routine vitamin D supplementation during pregnancy are established in Poland.

Since the collection of our data regarding preterm infants born at less than 32 weeks of gestation, several new studies have emerged in the literature. Interestingly, despite the theoretical preference for the chromatographic “gold standard” (LC-MS/MS), the majority of recent publications continue to utilize the automated electrochemiluminescence immunoassay (ECLIA) method for 25OHD determination, mirroring our own methodological approach [9,60]. However, while contemporary researchers have predominantly focused on postnatal supplementation strategies and immediate neonatal outcomes, our study provides a distinct clinical advantage. Specifically, our work offers a highly meticulous analysis of the comprehensive maternal and environmental risk factors directly associated with the development of severe hypovitaminosis D in both mothers and their very preterm offspring at birth. Furthermore, in light of the now widespread implementation of multivitamin and DHA co-supplementation from early pregnancy in Poland, our study provides an objective baseline for future follow-up trials. This unique chronological perspective allows for the empirical verification of several outcomes and hypotheses established in our cohort. By serving as a rigorous reference point before the near-universal adoption of these intensive prenatal regimens, our findings offer critical insights into the shifting trends of maternal–fetal vitamin D dynamics over the last decade.

## 5. Conclusions

Our findings confirm the well-documented association between neonatal and maternal vitamin D status. Our study aligned with previous research demonstrating that maternal 25OHD status is the primary determinant of neonatal vitamin D levels.

In summary, our study demonstrates a high prevalence of vitamin D insufficiency among mothers and their preterm offspring, particularly during the winter season. Maternal 25OHD levels are significantly higher in summer, correlating with increased supplementation and a lower prevalence of deficiency among mothers. Analysis indicates that higher cord blood vitamin D levels depend on both the season and supplementation; however, dermatological guidelines limit endogenous synthesis, making year-round supplementation essential.

Because the lack of improvement in prenatal supplementation rates persists despite existing guidelines, a more effective translation of these recommendations into clinical practice is urgently required. To effectively protect this vulnerable maternal–neonatal population, we propose the following clinical recommendations:

Early Counseling: Pregnant women must be explicitly counseled on the critical necessity of consistent vitamin D supplementation starting from the first trimester.

Targeted Maternal Assessment: Selective monitoring of serum 25OHD levels could be considered on an individual basis to establish tailored, adequate vitamin D intake in pregnant women at elevated risk for preterm labor.

Cord Blood Testing: Umbilical cord blood 25OHD measurements in preterm newborns delivered before 32 6/7 weeks of gestational age may serve as a valuable clinical indicator to guide personalized neonatal dosing, particularly when maternal vitamin D status during pregnancy remains unknown. Crucially, utilizing umbilical cord blood for these laboratory assessments directly reduces the volume of blood that needs to be drawn from the newborn. For premature infants, a chromatographic “gold standard” (LC-MS/MS) method that excludes the C3-epimer should be used to measure vitamin D levels, as this provides the most accurate assessment of vitamin D status.

In the context of preterm birth, periodic epidemiological evaluations of vitamin D status within the Polish population could be beneficial every 5–10 years, allowing for the verification of previous hypotheses to continually improve therapeutic outcomes.

## Figures and Tables

**Table 1:** Clinical characteristics and determinants of maternal and cord blood 25OHD levels.

	*n* (%)	Cord Blood 25OHD Level (ng/mL) Median IQR	*p*-Value	Maternal 25OHD Level (ng/mL) Median IQR	*p*-Value
**Neonatal data**					
Gestational age					
22–28 GA	29 (42.0%)	21.00 (16.60–33.00)	0.48	20.65 (16.00–28.48)	0.96
29–32 GA	40 (58.0%)	26.50 (16.20–35.40)		21.00 (12.50–29.25)	
Birth weight					
<1000 g	28 (40.6)%	22.25 (16.90–33.50)	0.912		
≥1000 g	41(59.4%)	26.00 (15.00–34.00)			
SGA	3 (4.4%)	58.00 (18.00–59.00)	0.12	48.36 (16.00–48.36)	
AGA	66 (95.6%)	24.5 (16.00–34.00)		20.65 (14.70–29.00)	
Apgar score					
0–4	7 (10.2%)	33.00 (21.90–35.00)	0.30		
5–7	33 (47.8%)	27.00 (16.60–33.00)			
8–10	29 (42.0%)	21.00 (15.00–34.00)			
Sex					
Male	38 (55.1%)	26.00 (17.00–35.00)	0.33		
Female	31 (44.9%)	21.90 (15.00–33.00)			
Season of birth					
Summer	25 (36.2%)	32.00 (16.60–39.80)	0.12	20.00 (29.25–39.40)	0.0005
Winter	44 (63.8%)	22.10 (16.50–33.00)		18.50 (11.58–27.00)	
**Maternal data**					
Vitamin D supplementation					
No	32 (45.6%)	17.40 (14.00–27.00)	<0.01	16.75 (11.00–22.00)	0.0006
Yes	37 (54.4%)	32.40 (20.60–36.80)		27.00 (19.00–30.00)	
DHA supplementation					
No	33 (47.8%)	21.00 (16.00–32.00)	0.06	18.50 (13.00–22.40)	0.019
Yes	36 (52.2%)	27.75 (16.75–36.20)		27.00 (18.00–30.50)	
Age (years)					
<28	14 (20.0%)	15.00 (11.00–28.48)	0.38		
28–35	42 (60.0%)	27.25 (18.00–29.25)			
>35	14 (20.0%)	25.00 (13.00–29.00)			
Parity					
1st pregnancy	41 (58.6%)	26.00 (16.00–34.00)	0.90		
2nd or 3rd pregnancy	25 (35.7%)	22.90 (16.80–35.00)			
≥4th pregnancy	4 (5.7%)	24.95 (16.80–31.00)			
BMI (kg/m^2^)					
<19.8	15 (22.0%)	26.00 (18.00–29.00)	0.89	23.00 (18.00–29.00)	
19.8–26	36 (53.0%)	25.00 (13.5–29.63)		20.50 (13.5–29.63)	
>26–29	7 (10.3%)	19.80 (11.00–21.20)		18.00 (11.00–21.20)	
>29	10 (14.7%)	20.80 (13.00–30.00)		21.00 (13.00–30.00)	
Weight gain during pregnancy					
Too small	31 (45.6%)	27.00 (19.00–33.00)	0.44	22.40 (18.00–24.00)	0.14
Normal	23 (33.8%)	20.60 (15.00–36.00)		19.00 (11.00–30.00)	
Too large	14 (23.3%)	19.50 (13.00–35.80)		21.92 (11.16–28.48)	
Gestational hypertension					
No	52 (75.7%)	27.00 (18.00–34.50)	0.20	21.20 (16.80–29.20)	0.12
Yes	17 (24.3%)	19.80 (15.60–32.00)		16.50 (10.00–29.00)	
Dopegyt					
No					
Yes	13 (18.8%)				
Hypothyroidism					
No	57 (82.61%)	23.20 (16.60–33.00)	0.33	20.00 (14.70–27.10)	0.14
Yes	12 (17.39%)	30.35 (16.60–46.15)		28.87 (14.40–31.50)	

SGA: small for gestational age, AGA: appropriate for gestational age, DHA: docosahexaenoic acid, BMI: body mass index. For women with a pre-pregnancy BMI < 19.8 (underweight), appropriate weight gain was defined as 12.5–18 kg; for a BMI of 19.8–26 (normal weight), 11.5–16 kg; for a BMI of 26–29 (overweight), 7–11.5 kg; and for a BMI > 30, up to 7 kg.

**Table 2:** Maternal and neonatal vitamin D status on the day of birth.

	25OHD Level		
	≤20 ng/mL	>20–30 ng/mL	>30 ng/mL
Newborns, *n* (%)	26 (37.68%)	17 (24.64%)	26 (37.68%)
Summer, *n* (%)	9 (34.3%)	2 (11.8%)	12 (46%)
Winter, *n* (%)	17 (65.4%)	15 (88.2%)	14 (54%)
Mothers, *n* (%)	28 (40.58%)	30 (43.48%)	11 (15.94%)
Summer, *n* (%)	5 (17.9%)	8 (27%)	8 (72.7%)
Winter, *n* (%)	23 (82.1%)	22 (73%)	3 (27.3%)
Median (IQR) maternal vitamin D intake, IU/d	0.00 (0.00–198.4)	114.9 (0.00–797.5)	1180 (655.24–1400)

**Table 3:** Univariate linear regression analysis.

Obs.	Variable	β Estimate	Lower CL	Upper CL	*p*
1	Maternal 25OHD level	0.83670	0.61558	1.05782	<0.0001
2	Maternal r 25OHD	0.00005398	0.00002914	0.00007883	<0.0001
3	Maternal 25OHD_day	0.01095	0.00584	0.01605	<0.0001
4	Weeks of gestation	0.70686	−0.67278	2.08651	0.3101
5	Birth weight	−0.00388	−0.01169	0.00394	0.3256
6	Birth weight pc	−0.12696	−0.24512	−0.00879	0.0356
7	Apgar score	−0.86514	−2.58734	0.85707	0.3195
8	Sex male	3.20747	−3.27295	9.68789	0.3267
9	Season of birth	−6.22318	−12.80543	0.35906	0.0635
10	Maternal supplementation _vitamin D Y/N	11.53601	5.87911	17.19291	0.0001
11	Maternal supplementation _DHA Y/N	6.03939	−0.29149	12.37028	0.0612
12	Prenatal steroids	3.05044	−5.48340	11.58428	0.4780
13	Maternal age	−0.01111	−0.59126	0.56903	0.9696
14	Maternal body weight gain	−0.05688	−0.66799	0.55424	0.8532
15	BMI	−0.21156	−0.92955	0.50642	0.5583
16	Maternal hypertension	−2.56776	−10.07682	4.94130	0.4972

**Table 4:** Multiple linear regression analysis of independent factors responsible for neonatal 25OHD levels.

Obs.	Variable	β Estimate	Lower CL	Upper CL	*p*
1	Maternal 25OHD level	0.76114	0.52132	1.00095	<0.0001
2	Male sex	6.35755	1.77365	10.94145	0.0073
3	Maternal supplementation yes/no	5.44695	0.55386	10.34005	0.0297

## Data Availability

The raw data supporting the conclusions of this article will be made available upon request at: mskrzypek@sum.edu.pl.
